# Role of Rho/MRTF in Aggressive Vemurafenib-Resistant Murine Melanomas and Immune Checkpoint Upregulation

**DOI:** 10.3390/ijms241813785

**Published:** 2023-09-07

**Authors:** Bardees M. Foda, Richard R. Neubig

**Affiliations:** 1 Department of Pharmacology and Toxicology, Michigan State University, East Lansing, MI 48823, USA; fodabard@msu.edu; 2 Molecular Genetics and Enzymology Department, National Research Centre, Dokki 12622, Egypt; 3 Nicholas V. Perricone, M.D. Division of Dermatology, Department of Medicine, Michigan State University, East Lansing, MI 48823, USA

**Keywords:** melanoma, resistance, immune checkpoint, BRAF, RhoGTPase

## Abstract

Cutaneous melanoma is the deadliest skin cancer. Most have Ras-MAPK pathway (BRAF^V600E^ or NRAS) mutations and highly effective targeted therapies exist; however, they and immune therapies are limited by resistance, in part driven by small GTPase (Rho and Rac) activation. To facilitate preclinical studies of combination therapies to provide durable responses, we describe the first mouse melanoma lines resistant to BRAF inhibitors. Treatment of mouse lines, YUMM1.7 and YUMMER, with vemurafenib (Vem), the BRAF^V600E^-selective inhibitor, resulted in high-level resistance (IC_50_ shifts 20–30-fold). Resistant cells showed enhanced activation of Rho and the downstream transcriptional coactivator, myocardin-related transcription factor (MRTF). Resistant cells exhibited increased stress fibers, nuclear translocation of MRTF-A, and an increased MRTF-A gene signature. Pharmacological inhibition of the Rho/MRTF pathway using CCG-257081 reduced viability of resistant lines and enhanced sensitivity to Vem. Remarkably, co-treatment of parental lines with Vem and CCG-257081 eliminated resistant colony development. Resistant cells grew more slowly in vitro, but they developed highly aggressive tumors with a shortened survival of tumor-bearing mice. Increased expression of immune checkpoint inhibitor proteins (ICIs) in resistant lines may contribute to aggressive in vivo behavior. Here, we introduce the first drug-resistant mouse melanoma models for assessing combinations of targeted and immune therapies.

## 1. Introduction

Despite effective treatments for melanoma, drug resistance, both intrinsic and acquired, limits durable cures [1,2]. Growth of mutant BRAF^V600E/K^ melanoma tumors is sustained by constitutive activity of the MAPK pathway, and tumor growth is inhibited by targeted therapies such as vemurafenib, trametinib, and dabrafenib, but resistance inevitably develops. Similarly for immunotherapies, including anti-PDL1, anti-PD1, or anti-CTLA4 [3], intrinsic or acquired resistance is problematic. Combined treatments can partially overcome relapse or limited responses to monotherapy [4,5,6], however, a better understanding of resistance-triggering mechanisms and good models for their study will be needed to enhance the efficacy of current treatments. 

Reactivation of the MAPK pathway is a common acquired resistance mechanism [7,8,9], however, MAPK-independent resistance mechanisms also contribute significantly. RhoA, RhoC, and Rac1 regulate cell cytoskeleton and gene transcription [10,11] and are known to enhance melanoma migration, metastasis, and drug resistance. Enhanced activity of Rho GTPases may contribute to up to 50% of melanoma drug resistance [12,13,14]. The effects of Rho depend on gene transcription mechanisms involving serum response transcription factor (SRF) [11,15] and/or YAP/TAZ [16]. Rho-regulated SRF-mediated gene transcription depends on the myocardin-related transcription coactivators (MRTFA/B) [17], which physically bind to SRF on DNA to regulate gene transcription involved in cellular differentiation, cytoskeletal regulation, adhesion, migration, and proliferation. In human melanoma, we identified a key role for Rho and MRTF-A in drug resistance [13,18]. Clinical evidence supporting this model also includes high-Rho activities and increased MRTF gene signatures in melanoma tumors collected from patients relapsed on MAPK inhibitors [13]. This gains greater importance in light of the effect of Rho signaling in resistance to immune-checkpoint therapies [14].

In this report, we address the lack of murine melanoma models with BRAFi resistance. To accomplish this, we treated Yale University Mouse Melanoma (YUMM) lines with the BRAF inhibitor, Vem, in vitro to generate resistant cells. Two highly resistant lines derived from YUMM1.7 and its UV-irradiated counterpart YUMMER showed enhanced activity of the Rho/MRTF pathway. Furthermore, inhibition of MRTF signaling with CCG-257081 enhanced sensitivity to Vem and actually prevented the onset of Vem resistance. Also, the Vem–resistant lines showed an increased expression of immune checkpoint proteins and developed aggressive tumors in vivo, which grew much more rapidly than parental tumors in non-immunocompromised mice. These lines will permit exploration of mechanisms of resistance and preclinical testing of combined targeted therapies and immune checkpoint inhibitors.

## 2. Results

### 2.1. Generating Vem–Resistant Mouse Melanoma Lines

**Figure 1:**
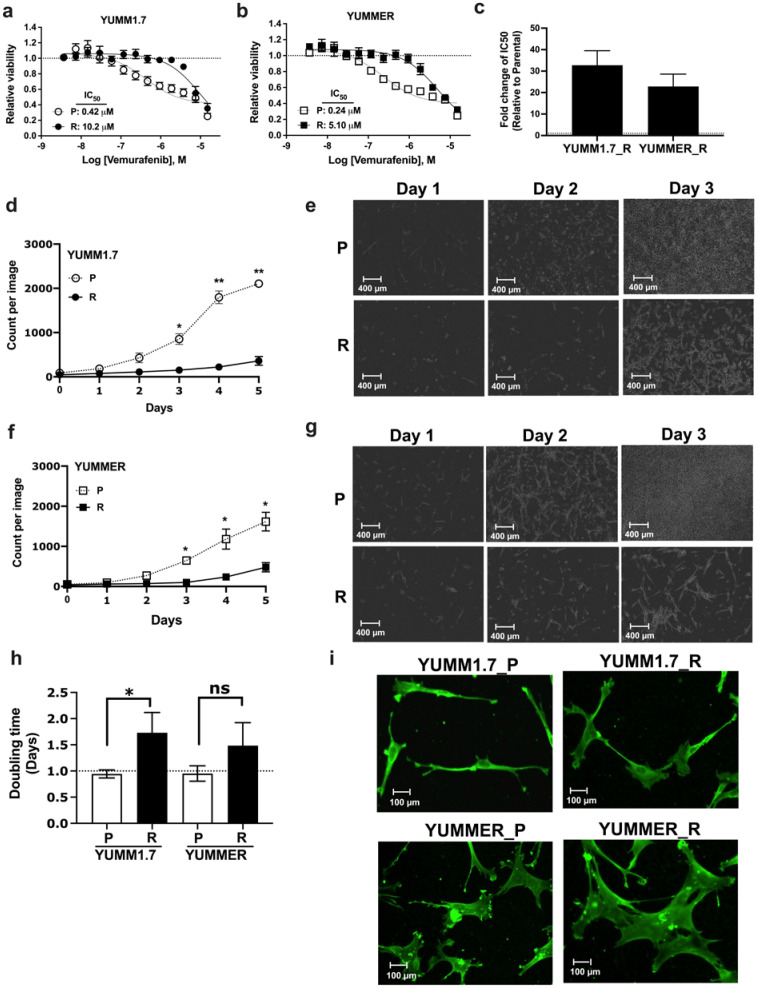
Generation of Vem–resistant YUMM lines. (**a**,**b**): Vem–sensitivity of BRAF^V600E^ cell lines (YUMM1.7 and YUMMER) before (P) and after (R) development of resistance to Vem. Cells were treated with increasing concentrations of Vem for 72 h in DMEM medium supplemented with 10% FBS. An ATP-based assay (see Section 4) was used to calculate cell viability relative to DMSO-treated cells. (**c**): Calculated fold-change in IC_50_ of Vem of resistant lines relative to parental cells. Data are mean ± SEM of five independent experiments. (**d**–**g**): Parental and resistant YUMM cells were cultured on a 24-well plate in medium with 10% FBS, and proliferation was tracked using the IncuCyte instrument phase module. (**d**,**f**): Cell count was determined using phase-object image analysis. (**e**,**g**): images captured using the 10× IncuCyte’s objective. (**h**): Doubling time was calculated using incuCyte data (see Section 4). Results are the mean ± SEM of three independent experiments. (**i**): Morphology of parental and resistant cells, immune-stained, fixed melanoma cells with vimentin antibody. ns, nonsignificant * *p* < 0.05; ** *p* < 0.01.

Using six different YUMM mouse melanoma lines [19,20], we measured their sensitivity to Vem. The lines harbor the most prevalent melanoma mutation, *Braf*^V600E^, and additional mutations that known to drive melanoma in human. The cells were then subjected to prolonged exposure to 5 mM Vem (Supplementary Figure S1a and Table S1). Two lines were intrinsically resistant (YUMM4.1 *Braf*^WT^ and YUMM1.G.1 *Braf*^V600E^) and showed no change in IC_50_ with Vem treatment. Two other *Braf*^V600E^ lines (YUMM3.3 and YUMM5.2) showed modest resistance (4- and 8-fold increases in IC_50,_ respectively). Two *Braf*^V600E^ lines (YUMM1.7 and YUMMER) showed high initial Vem–sensitivity (IC_50_ 0.42 and 0.24 mM, respectively) and, after 6–8 weeks in 5 mM Vem, produced high-level resistant (R) lines (fold-change in IC_50_ from parental of 33 ± 6.7 for YUMM1.7_R and 23 ± 5.7 for YUMMER_R (Figure 1a–c). Further studies focused on these two lines are needed.

### 2.2. Resistant Cells Have Reduced Proliferation Rates and Altered Morphology

Resistant melanoma cells often show reduced proliferation rates [21,22]. We observed that the Vem–resistant murine melanoma lines grew more slowly in real-time analysis in an Incucyte imaging system vs. the parentals (Figure 1d–g). This was also confirmed by cell counts (Figure 1h) with doubling times of YUMM1.7 (P: 22.6 h vs. R: 41.5 h) and YUMMER (P: 22.8 h vs. R 35.6 h). While the doubling time of the YUMMER line was not statistically and significantly different between resistant and parental, there is a trend of slower proliferation of the former. The lack of significance might be due to a more heterogenous cell population with various slow cell cycling rates that might lead to greater variability in the rate cell proliferation. Immunostaining of Vimentin to define their architecture revealed that the resistant cells were larger and had a more mesenchymal-like cell morphology (Figure 1i). Resistant YUMM cells had an increased fraction of flat, poly-polar cells with a larger cell surface area (YUMM1.7, 1.3 ± 0.08 mm^2^ for P and 1.7 ± 0.09 for R; YUMMER, 2.0 ± 0.1 for P and 4.6 ± 0.2 for R (Supplementary Figure S1b).

### 2.3. Vem Insensitivity of Resistant Cells Is Independent of MAPK Reactivation

**Figure 2:**
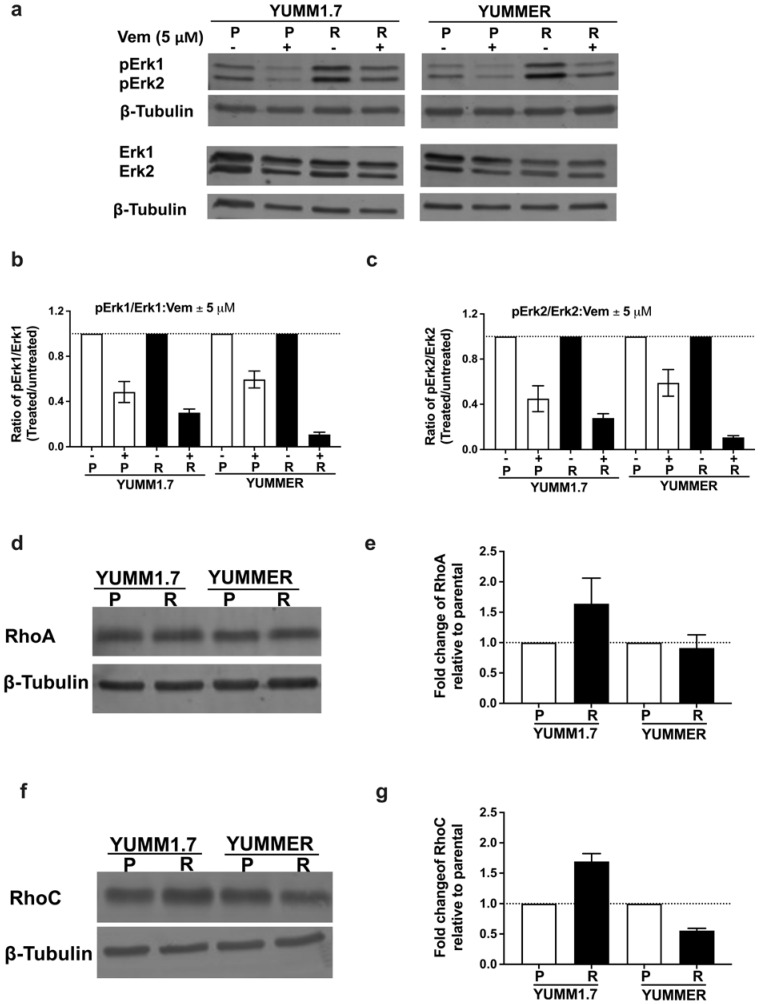
Vem–resistance is independent of MAPK pathway reactivation but is associated with upregulation of Rho proteins. (**a**): Western blot analysis of phosphorylated Erk proteins (pErk1/2) YUMM in cells treated for 48 h with 5 µM Vem or DMSO (−). (**b**,**c**): Quantification of pERK/ERK: the ability of 5 mM Vem to inhibit MAPK activity was assessed from the ratio of band densities for p-Erk1/2 vs. Erk1/2. Resistant lines showed similar or greater Vem–mediated inhibition of MAPK activity than parental cells. Results represent mean ± SEM from three independent experiments. (**d**–**g**): Expression of Rho A and Rho C proteins was assessed by immunoblotting whole cell lysates (30 µg total protein) with values normalized to β-tubulin. The quantified results (**e**,**g**) represent the mean ± SEM of four independent experiments.

To evaluate the contribution of MAPK reactivation to Vem–resistance, we measured levels of phosphorylated Erk1/2 (pErk1/2) after incubating parental or resistant YUMM cells with 5 µM Vem (Figure 2a–c). Immunoblot analyses of YUMM1.7_P and YUMMER_P cells showed reduced phosphorylation of Erk1/2 when exposed to Vem. Surprisingly, the resistant cells showed an even greater reduction in pErk1/2 phosphorylation upon exposure to Vem than the parental lines. Therefore, reactivation of the MAPK pathway does not appear to be the main mechanism of resistance.

### 2.4. The Rho/MRTF Pathway Is Activated in Vem–Resistant YUMM Cells

**Figure 3:**
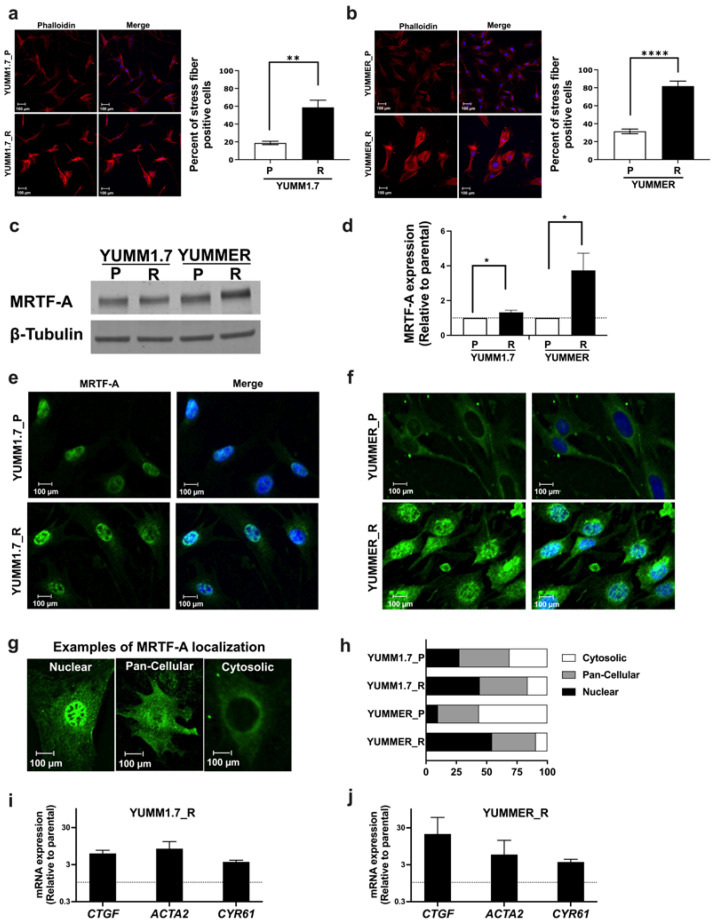
Vem–resistance is associated with enrichment of markers for activation of the Rho/MRTF pathway. (**a**,**b**): Fixed cells were stained with Rhodamine Phalloidin and DAPI. Actin stress fiber-positive cells (minimum of 200 cells per sample) were counted manually (see criteria in Section 4). Statistical analysis was performed using unpaired *t*-test to compare parental and resistant cells. The results represent the mean ± SEM of three biological replicates; the scale bar is 100 μm. (**c**,**d**): Western blot analysis of MRTF-A protein from four independent experiments; Relative expression was normalized to β-tubulin and then to the corresponding parental cells. (**e**,**f**): Nuclear localization of MRTF-A was assessed by indirect immunofluorescence. Representative images from three independent experiments are shown; the scale bar is 100 μm. (**g**): Examples of Nuclear, Pan-Cellular, and Cytosolic localization are depicted. (**h**): Quantification of the MRTF-A localization from panels (**e**,**f**). (**i**,**j**): qRT-PCR of genes regulated by MRTF pathway. The mRNA levels were normalized to the reference gene *Tbp*, and fold-change was calculated relative to the corresponding parental line. * *p* < 0.05; ** *p* < 0.01; **** *p* < 0.0001.

Since activation of Rho GTPases and downstream transcription factor signaling through MRTF or YAP is a common mechanism for melanoma resistance without MAPK-reactivation; therefore, we asked if Rho signaling was increased in our resistant mouse lines. First, we measured expression of RhoA family members (Figure 2d–g and Supplementary Figure S1c,d). YUMM1.7_R had modest increases in RhoA (64%) and RhoC (69%), while the YUMMER_R line showed small decreases in Rho protein levels: RhoA (10%) and RhoC (44.3%). As a measure of a downstream Rho mechanism, we assessed actin stress fiber staining with rhodamine-phalloidin. Stress fibers were defined as cells with at least one wide phalloidin-stained fiber spanning more than 90% of the entire cell area. Cortical F-actin was not considered to be related to Rho signaling (Supplementary Figure S2). The fraction of cells positive for actin stress fibers was significantly increased in resistant lines vs. the parental lines (YUMM1.7: 18.7 ± 4.2% for P, 58.7 ± 18.1% for R; YUMMER: 31.4 ± 5.9% for P, 81.8 ± 12.6% for R, Figure 3a,b).

### 2.5. The MRTF Pathway Is Upregulated in Resistant YUMM Cells

Activation of Rho induces actin remodeling which drives MRTF-mediated gene expression. To explore activities of the MRTF pathway, we first asked if resistant cells had altered expression or nuclear localization of MRTF-A. Relative to isogenic parentals, YUMMER_R had a 3.7-fold increase in MRTF-A protein levels, and YUMM1.7_R showed a 33% increase (Figure 3c,d). To further assess activation of MRTF-A, we measured nuclear localization of MRTF-A. Immunofluorescence staining displayed increased nuclear localization MRTF-A in resistant cells, particularly for YUMMER_R (9.9% for P, 54.5% for R, Figure 3e–h). Cell fractionation experiments confirmed the immunolocalization results; nuclear lysates of resistant cells contained higher levels of MRTF-A than their parental counterparts (Supplementary Figure S3).

Furthermore, we tested whether the increased nuclear localization of MRTF-A was associated with upregulated gene expression. Increased mRNA levels were found in the resistant lines for genes known to be direct targets [23]: alpha smooth muscle actin (*Acta2*), connective tissue growth factor (*Ctgf*), and Cysteine-rich angiogenic inducer 61 (*Cyr61*) (Figure 3i,j). Therefore, Vem–resistant YUMM cells showed increases in multiple measures of MRTF activation and MRTF-regulated gene transcription.

### 2.6. Inhibition of the Rho/MRTF Pathway Enhances Vem Sensitivity

**Figure 4:**
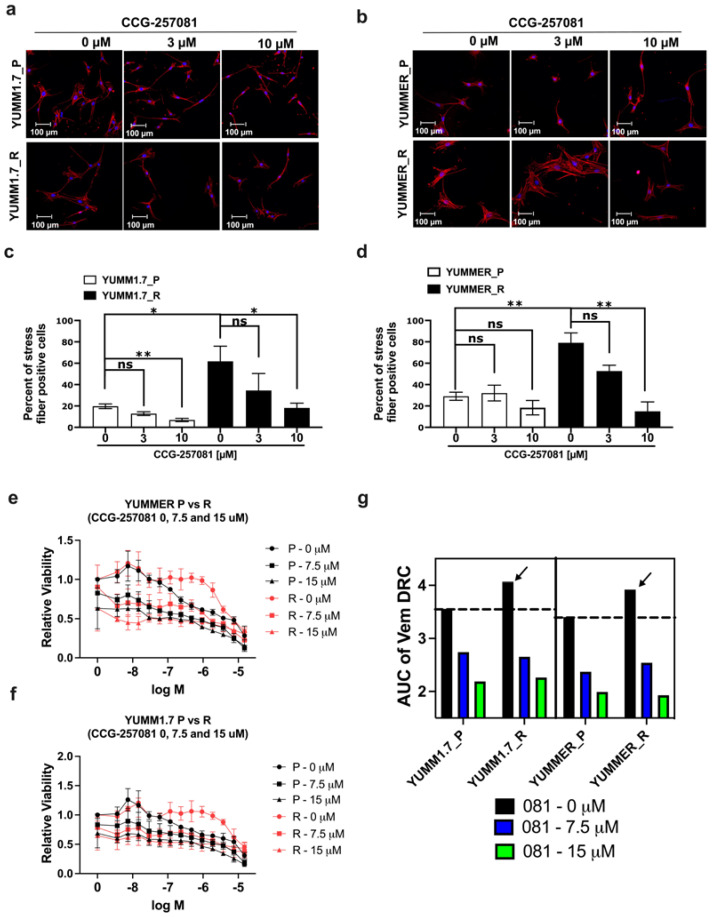
Inhibiting Rho/MRTF pathway diminished actin stress fibers and reversed the Vem–insensitivity of resistant YUMM lines. (**a**,**b**): YUMM cells were treated with 3 or 10 µM CCG-257081 or DMSO control for 24 h, then fixed and stained with Rhodamine Phalloidin and DAPI. (**c**,**d**): Actin stress fibers were quantified as described in Section 4. Statistical analysis was performed using unpaired *t*-tests. * *p* < 0.05; ** *p* < 0.01; ns, not significant. Representative images are from three independent biological replicates; the scale bar is 100 μm. (**e**,**f**): The Vem sensitivity of Parental and Vem–resistant cells was tested with and without CCG-257081. Cells were treated with Vem and CCG-257081 at the indicated concentrations for 72 h, then viability was measured with CellTiter-Glo. Viability data were normalized to untreated cells (in the presence of DMSO). Data were fit to 3-parameter inhibition curves (i.e., Hill slope = 1) in GraphPad Prism v.10 software with the bottom parameter constrained to be greater than zero and fitted parameters are shown in Table S2. (**g**): IC_50_ values were difficult to determine accurately, so an AUC analysis was done examine Vem–sensitivity. With DMSO vehicle, the two resistant lines (arrows) showed an AUC value for the Vem concentration response curve at least 0.5 units greater than that of the parental, Vem–sensitive, cells. CCG-257081 at 7.5 and 15 mM eliminated the AUC difference between P and R cell lines. Data are averages from three independent experiments.

We sought to determine whether activation of the MRTF pathway contributes to Vem–resistance. Using CCG-257081, an MRTF-pathway inhibitor (MRTFi) acting downstream of ROCK [24,25] we tested effects of the compound on measures of Rho/MRTF signaling and cell sensitivity to Vem. Treating YUMM1.7_R and YUMMER_R with 10 µM of CCG-257081 for 24 h significantly reduced actin-stress fibers (Figure 4a–d). CCG-257081 (10 µM) also significantly reduced stress fibers in the parental YUMM1.7_P cells, but not in the YUMMER_P cells.

Since Rho/MRTF pathway signaling was increased in resistant melanoma cells, we asked if we could reverse Vem–resistance of stable resistant cells using our MRTF pathway inhibitor, CCG-257081. We performed “cross-dose–response curves” as previously performed with human melanoma cell lines [26]. The resistant cells showed a 0.5 unit increase in the area under the curve (AUC) for the Vem concentration response curve (arrows, Figure 4g). Adding 7.5 or 15 mM CCG-257081 modestly reduced the viability of the parental and resistant cell lines but it also eliminated the difference in the Vem concentration response curves between parental and resistant lines (Figure 4e,f). The MRTF pathway inhibitor also equalized the Vem AUC values between parental and resistant YUMM1.7 and YUMMER lines (Figure 4g).

**Figure 5:**
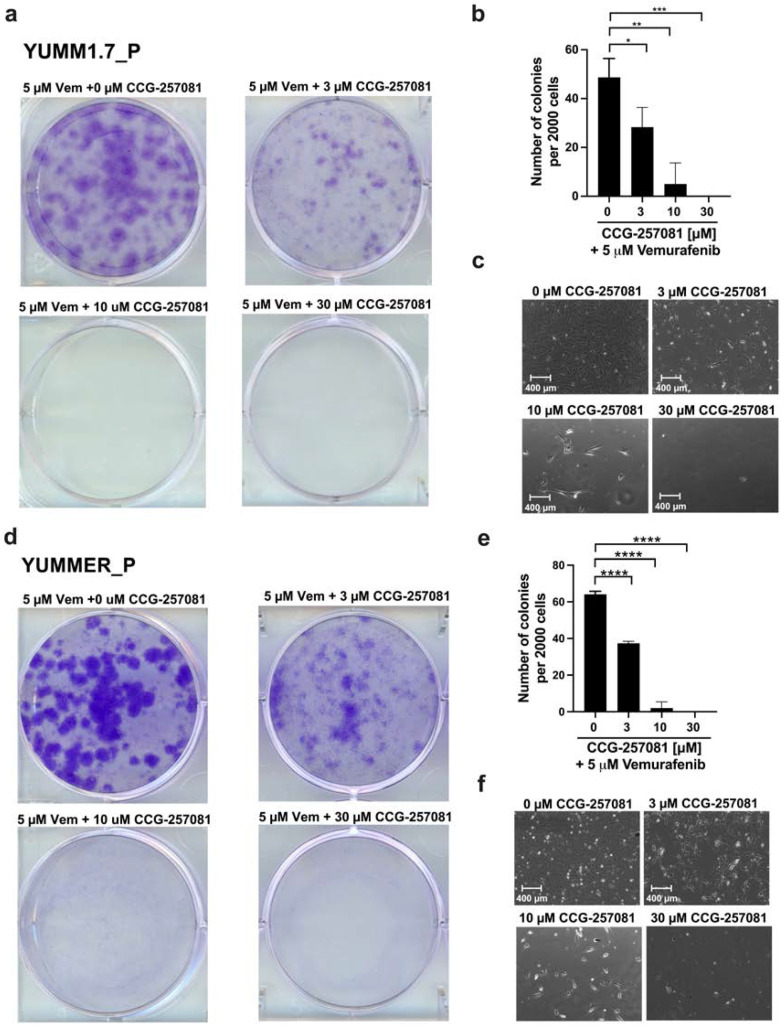
Inhibition of Rho/MRTF pathway prevents the development of Vem resistance. To mimic the method used in the initial development of the Vem–resistant cell lines, colony formation assays were done on YUMM1.7_P (**a**–**c**) and YUMMER_P (**d**–**f**) cells, cultured in the presence of 5 µM Vem and increasing concentrations of CCG-257081, as indicated. Colonies were stained with crystal violet (**a**,**d**), and the number of colonies was determined using ImageJ (**b**,**e**). Images of cells within one colony were captured with a light microscope before staining (**c**,**f**). Results are the mean of three independent experiments, * *p* < 0.05; ** *p* < 0.01; *** *p* < 0.001; **** *p* < 0.0001.

We then asked whether CCG-257081, by inhibiting Rho/MRTF signaling, can actually prevent resistance development to Vem. To test this, we performed a colony formation assay using parental YUMM cells in the presence of 5 µM Vem co-treated with increasing concentrations of CCG-257081. We monitored development of colonies that are stably resistant to Vem. Interestingly, co-treatment sharply diminished the development of resistant cells (Figure 5). These results imply that Rho/MRTF activation is a substantial factor in the development of Vem–resistance and that suppressing this activity may abort drug resistance. This could represent a valuable combination treatment to mitigate targeted therapy resistance.

### 2.7. Effect of Targeted Therapy Resistance on Immune Checkpoint Gene Expression

**Figure 6:**
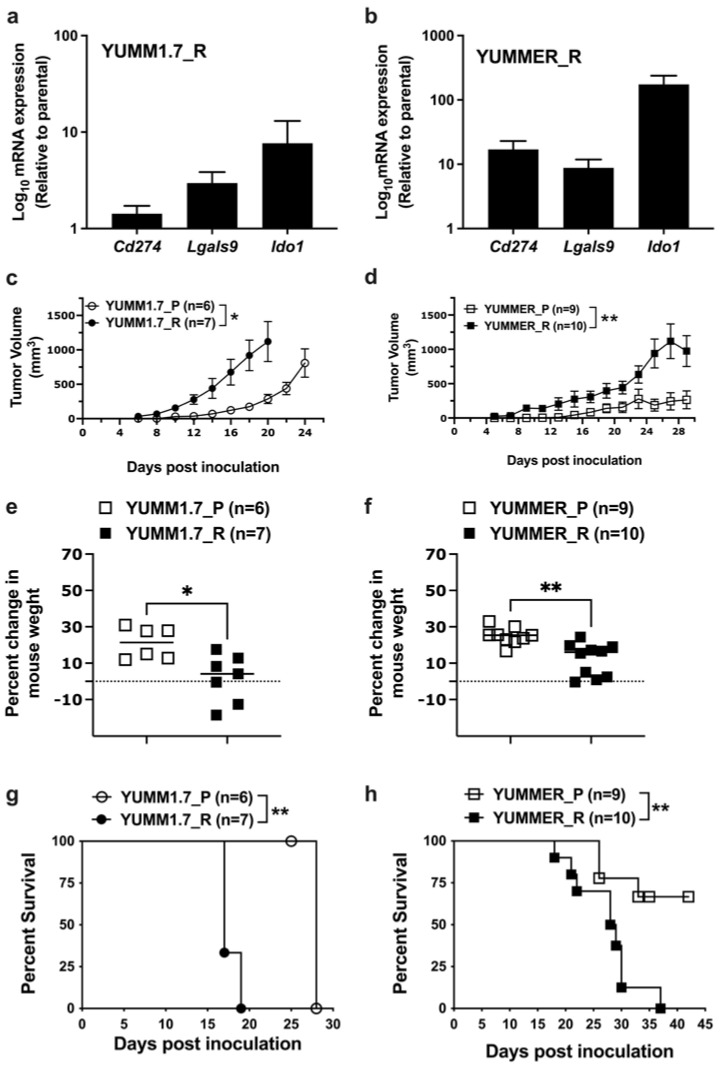
Vem resistance upregulates gene expression of ICIs and enhances the growth of YUMM tumors in vivo. (**a**,**b**): mRNA levels of ICIs (*Cd274*, *Lgals9*, and *Ido1*) in YUMM1.7_R and YUMMER_R cells relative to the corresponding parental line. The mRNA levels were normalized to the reference gene *Tbp*, and fold-change was calculated relative to the corresponding parental line. (**c**,**d**): Tumor growth in male C57BL/6 mice injected subcutaneously with 1 × 10^6^ of YUMM1.7_P or YUMM1.7_R (**a**) and YUMMER_P or YUMMER_R (**b**). Tumor growth was monitored every two days throughout the entire course of the experiment and mice were sacrificed at the approved humane endpoint. (**e**,**f**): Change of body weight of mice at endpoint relative to the weight before cell injection. (**g**,**h**): Percentage of surviving mice. The survival curve endpoints were body weight loss (<10%), tumor ulceration, or difficulty ambulating. A 2-way ANOVA was used for the tumor size, log-rank test for survival curves, and *t*-test for body weight changes. * *p* < 0.05; ** *p* < 0.01.

Cancer cells possess several mechanisms to help tumors escape the immune system, such as recruiting immunosuppressive leukocytes or transmitting inhibitory signaling to activated immune cells [27]. Cancer cells downregulate activities of immune cells by expressing inhibitory immune checkpoint proteins to escape immune mechanisms [28]. Evidence suggests that initiating treatment of BRAF^mut^ melanoma with BRAF inhibitors. prior to immune checkpoint treatments, worsens the outcomes of those treatments [29]. This suggests that targeted therapy resistance may induce gene expression changes that interfere with Immune checkpoint therapy (ICT). Thus, we tested the gene expression of some ICIs, including programmed cell death ligand 1 (PDL-1—gene name *Cd274*), indoleamine 2,3-dioxygenase 1 (IDO1), and galectin 9. Resistant YUMM cells upregulated the expression of genes encoding PDL1 (*Cd274*), galectin9 (*Lgals9)*, and IDO1 (*Ido1*) (Figure 6a,b). The YUMMER_R line showed significantly greater increases (10–200-fold), which makes YUMMER_R a strong candidate to test combination therapies to overcome immune resistance. While both lines show evidence for increases in mRNA expression of the checkpoint inhibitor genes, the mechanism of the difference between the magnitude of effects in YUMMER_R vs. YUMM1.7_R is not clear.

### 2.8. Enhanced In Vivo Tumor Growth by Vem–Resistant Cell Lines

Given that resistant cell lines proliferated much more slowly in 2D tissue cultures, we wanted to know how they would behave in vivo. Previous studies showed that activated Rho/MRTF can enhance melanoma progression and metastasis. We injected 10^6^ cells of YUMM1.7_P, YUMM1.7_R, YUMMER_P, or YUMMER_R lines subcutaneously in wildtype C57BL/6 mice. Despite slower growth in 2D culture, resistant YUMM cells resulted in significantly larger tumors at earlier times than parental tumors (Figure 6c,d). Mice injected with parental cells were in good health, gaining weight and remaining active. However, mice inoculated with resistant cells gained less body weight (Figure 6e,f), had more aggressive tumors, and some ultimately developed skin ulcers. This was reflected in survival curves to a composite endpoint (>10% body weight loss, ulceration, or difficulty ambulating). There was reduced or delayed impairment for mice with the parental tumors vs. the resistant ones (Figure 6g,h). Thus, resistant YUMM cells with activated Rho/MRTF represent potential melanoma models with aggressive tumors that can be used in non-immunocompromised mice to facilitate studies of immune mechanisms in vivo.

## 3. Discussion

This report presents the first mouse melanoma models with Vem resistance to permit mechanistic, preclinical studies of double or triple treatments with targeted therapies and immunotherapy. Resistant cells formed rapidly growing aggressive tumors in vivo. They showed increased activation of the Rho/MRTF pathway and enhanced immune checkpoint gene expression, but no reactivation of MAPK signaling. The Rho/MRTF pathway inhibitor CCG-257081 enhanced the effectiveness of Vem and, strikingly, prevented cells from acquiring Vem–resistance.

The resistant YUMM cells grow slowly in vitro, which could be due to enrichment of slow cycling cells with stem cell characteristics [30,31]. Such cells are insensitive to drug treatment and can exit from quiescence into dysregulated growth, driving dormancy and metastatic dissemination [32]. Their dynamic nature also accelerates metabolic and epigenetic alterations, resulting in tumor heterogeneity [22,33]. These features may contribute to the aggressiveness in vivo of the resistant YUMM tumors in our study.

Understanding resistance mechanisms can help identify potential therapeutics to prevent or reverse resistance. Recent clinical practice has moved from Vem monotherapy to the combination of BRAF and MEK inhibitors. We previously showed that the mechanism explored here, Rho and MRTF activation, also results in resistance to MEK inhibitors such as trametinib [26]. This suggests that the results described here will likely be relevant in dual therapy resistance as well as for Vem–resistance alone. Approximately 50% of resistant melanomas lack MAPK-reactivation and a similar fraction show increased Rho/MRTF signaling [13,34,35], as we see here. This drives multiple cell functions such as gene expression, cell proliferation, adhesion, migration, and cytoskeleton rearrangement [36,37,38] and dysregulated Rho/MRTF promotes cancer progression and metastasis [39,40,41,42]. Inhibition of MRTF signaling in human melanoma suppressed RhoC-mediated lung metastases [43] and enhanced in vitro sensitivity to targeted therapies [13,26], behavior similar to our results here.

Enhanced gene expression downstream of Rho/MRTF (e.g., *Ctgf*, *Acta2*, and *Cyr61*) in the resistant cells agreed with increased nuclear translocation of MRTF-A. CTGF promotes cell proliferation, adhesion, and migration [44,45,46]. CTGF facilitates metastasis [47], and reducing its expression diminished bone and brain metastases in a melanoma mouse model [48,49]. *ACTA2* encodes α-smooth muscle actin, which contributes to cytoskeletal dynamics and cell migration [50]. Interestingly, melanomas with elevated levels of ACTA2 were resistant to ICT [51]. This may be due to altered ICI expression, as observed here in the resistant parental YUMM1.7 and YUMMER or to changes in protein localization [19].

The Rho/MRTF pathway Inhibitor CCG-1423 and related compounds have been shown to reduce metastasis [43,52] and reverse resistance in multiple cancer models [18,53,54]. In addition to inhibiting Rho/MRTF-regulated gene transcription, these compounds also bind to the redox-regulated protein pirin [55], which has also been implicated in melanoma [56]. Pirin contributes to tumorigenesis and progressive malignancy of many tumors [56,57,58]. Pirin is implicated in several cellular activities including cell cycle and inflammatory responses, and has been suggested to be a marker of melanoma prognosis [59]. The role of pirin in Rho/MRTF signaling should be determined.

A key observation in this study is that addition of CCG-257081 in vitro prevented the formation of Vem–resistant colonies. This may be related to the synergistic effects of the compound to promote apoptosis with Vem, as previously reported in NRAS mutant human melanoma lines [26]. Alternatively, CCG-257081 could independently target a pre-resistant cell population [60]. Pre-resistant cells are rare in Vem–naïve melanomas, which may explain the minimal effect of CCG-257081/Vem cotreatment on the viability of parental cells compared to their resistant counterparts. Prevention of Vem–resistance by CCG-257081 also suggests that the Rho/MRTF pathway may be a main driver of Vem–resistance in resistant YUMM cells.

The interaction of targeted drugs and immunotherapy represents a critical focus in the development of highly effective melanoma therapies. Immunotherapy given after targeted therapy is less effective [61], but triple-therapy with BRAF/MEK inhibitors and immunotherapy agents is showing promise [62]. Interfering with the Rho/MRTF mechanism, as shown here, may represent an alternative approach to prevent the development of resistance to both targeted therapy and immune-checkpoint treatments and may enhance clinical responses both before and after resistance development. The availability of these mouse melanoma models that can be used in non-immunocompromised mice, especially the YUMMER_R resistance model that mimics human melanoma in having a high-mutational burden, will facilitate the investigation of new drug/immune-therapy combinations for aggressive and resistant melanomas.

## 4. Materials and Methods

### 4.1. Cell Culture and Selection of Vem–Resistant Populations

YUMM1.7 (SCC227) and YUMMER (SCC243) lines from Millipore Sigma (Burlington, MA, USA); YUMM1.G1 (CRL-3363), YUMM3.3 (CRL-3365), YUMM4.1 (CRL-3366), YUMM5.2 (CRL-3367) from ATCC (Manassas, VA, USA) were maintained in DMEM-F12 medium (ATCC (Manassas, VA, USA), 30-2006) supplemented with 10% FBS (Gibco (Waltham, MA, USA) #10437-028), 1% NEAA (Gibco, 11140-50), and 1% pen-Strep (ThermoFisher (Waltham, MA, USA), 15140122). For generating Vem–resistant lines, cells were seeded at ~20% confluence and allowed to adhere overnight, then cultured in medium containing 5 µM Vem (AmBeed (Arlington Heights, IL, USA), A116840) and refreshed every 2–3 days. Stably Vem–resistant cells reached a confluence after two months. Generated Vem–resistant cells were named YUMM1.7_R and YUMMER_R to be distinguished from their isogenic parentals, YUMM1.7_P and YUMMER_P, respectively.

### 4.2. Cell Viability

Cells were seeded into 384-well tissue culture plates (PerkinElmer (Waltham, MA, USA), #6007689) at a density of 1000 cells overnight. On the next day, increasing concentrations of drugs were added using a pin tool (~150 nL) and incubated for 72 h. To measure cell viability, CellTiter-Glo (Promega (Madison, WI, USA), G7573) was added, and plates were read on a Bio-Tek Synergy Neo-plate reader according to the manufacturer’s protocol. Cell viability readings were normalized to DMSO-treated cells. Data were plotted by GraphPad Prism (San Diego, CA, USA) using drug dose–response curves, and IC_50_ was calculated.

### 4.3. Compounds and Antibodies

Vem (AmBeed Inc. A116840) and CCG-257081 (synthesized in the MSU Medicinal Chemistry Core) were stored as 10 mM stocks in DMSO. MRTF-A (Proteintech (Rosamond, IL, USA), 21166-1-AP), RhoA (2117S), RhoB (2098S), RhoC (3430S), Erk1/2 (9102), pErk1/2 (4370S), and beta-tubulin (2146S) were purchased from Cell Signaling. Donkey anti-Rabbit800 (C926-32213) and Donkey anti-Rabbit680 (926-68073) immunoblotting secondary antibodies were purchased from LI-COR: Alexa Fluor goat anti-rabbit488 (#A11034) and donkey anti-Rabbit594 (A11037) were purchased from Invitrogen (Waltham, MA, USA).

### 4.4. RNA Extraction, cDNA Synthesis, and qRT-PCR Analysis

Cells were cultured and treated as indicated in each experiment. Total RNA was isolated using RNeasy Plus Kit (Qiagen (Germantown, MD, USA) #74134), as recommended by the manufacturer. cDNA was synthesized using the High-Capacity cDNA RT kit (ThermoFisher #4368814), according to the manufacturer’s guidelines. qPCR was performed using the SYBR Green PCR Master Mix (ThermoFisher #4309155) on the Applied Biosystems QuantStudio 7 Flex Real-Time PCR. qPCR primers were designed using the Harvard Primer Bank tool (https://pga.mgh.harvard.edu/primerbank/ (accessed on 29 November 2020)) and purchased from Integrated DNA. The primers used in the study included: *Tbp* Forward (3′-GCAATGTCTAACGGGGTTTACG-5′), *Tbp* Reverse (3′-TAGAGGTGTGCTGGACACTAC-5′); *CTGF* Forward (3′-CTGCAGACTGGAGAAGCAGA-5′); *CTGF* Reverse (3′-GATGCACTTTTTGCCCTTCTT-5′); *CYR61* Forward (3′-TAAGGTCTGCGCTAAACAACTC-5′), *CYR61* Reverse (3′-CAGATCCCTTTCAGAGCGGT-5′).

### 4.5. Western Blot Analysis

Adherent cells were cultured and treated as indicated. Cells were lysed on ice using lysis buffer (20 mM tris-HCl, pH7.5; 150 mM NaCl; 1 mM NaF; 1 mM ETDA; 0.5% NP40; 0.5 mM DTT) and supplemented with a protease inhibitor (Thermo Scientific# A32961 (Waltham, MA, USA)). Ice-cold, whole-cell lysates were sonicated gently with a probe sonicator. An equivalent amount (~30 µg) of each cell lysate was boiled in an SDS-loading buffer for 10 min. Samples were loaded onto a 10% or 12% polyacrylamide gel and transferred to the Immobilon-FL PVDF Membrane (Millipore Sigma, #IPFL00010). Membranes were blocked in Intercept LI-COR blocking buffer (PBS:927-70001 or TBS: 927-60001) and then incubated with primary antibody overnight at 4 °C. Washed membranes were incubated with the appropriate secondary antibody for 1 h at RT. The immunoblot membrane was washed, dried, and imaged on an LI-COR Odyssey FC imaging system. For determining nuclear enrichment of MRTF-A, cells were lysed using Cytoplasmic cell fractionation buffer (20 mM HEPES, pH 7.4; 10 mM KCl; 2 mM MgCl2; 1 mM EDTA; 1 mM EGTA, 1 mM DTT) and supplemented with the protease inhibitor. The ice-cold cell lysate was centrifuged to separate the cytoplasmic, mitochondria, and membrane fractions (soluble fraction) from nuclei (insoluble fraction). The soluble fraction was further spun to isolate the cytoplasmic fraction. The nuclei that were lysed in RIPA buffer (Boston Bioproducts (Milford, MA, USA) #BP-115-5X) were supplemented with the protease inhibitor and gently sonicated. An equivalent amount (~20 µg) of each fraction was boiled in an SDS-loading buffer for 10 min and loaded onto a 4–12% gradient polyacrylamide gel; then, the protocol was followed as described above.

### 4.6. Fluorescence Microscopy

#### 4.6.1. Immunofluorescence

YUMM cells were allowed to adhere onto fibronectin-coated coverslips overnight. Cells were fixed with 3.7% formaldehyde for 15 min at RT and then blocked and permeabilized in PBS containing 2% BSA and 0.1% Triton for 1 h at RT. Cells were incubated with primary antibody, MRTF-A, or Vimentin, at 1:200 titer overnight at 4 °C. The compatible secondary antibody was added at 1:15,000 for 1 h at RT. Coverslips were mounted onto microscopic slides using ProLong Gold Antifade/DAPI (ThermoFisher, P36935). Slides were imaged on a NikonTE2000-U Inverted Microscope (Tokyo, Japan).

#### 4.6.2. Staining Actin Stress Fibers

Cells were prepared as described above. To visualize F-Actin, cells were stained for 1 h at RT with Rhodamine Phalloidin (Cytoskeleton Inc. (Denver, CO, USA) PHDR1). Stress fiber analysis was performed by manual counting of at least 200 cells from each biological replicate. Criteria for detecting stress fibers included cells with at least one thick actin filament spanning more than 90% of the cell length, excluding those cells with only cortical actin.

### 4.7. Incucyte Live-Cell Imaging

IncuCyte S3 platform from Sartorius was used to monitor cell proliferation. About 5000 Cells were seeded in 24-well plates. Cells were scanned using a phase contrast channel. Images were captured every 15 min for 5 days using a 10× objective. Images captured for each line were analyzed by IncuCyte software (version: 2021C). The parameters were set to count each cell as one object considering size, boundaries, and shape. Proliferation curves were plotted using data collected from captured images. An exponential growth curve was used to calculate the doubling time.

### 4.8. In Vitro Clonogenicity Assay

Exponentially growing YUMM1.7_P and YUMMER_P cells were harvested. About 2000 cells were plated into a 6-well plate and left to adhere overnight. On the next day, drug treatment was applied (5 µM Vem ± 3 µM or 10 µM of CCG-25081). Plates were monitored until Vem–treated cells formed sufficiently large colonies (about 50 cells per colony), which took about 15 days. Colonies were washed in PBS and stained with a fixation-staining solution containing 3.7% formaldehyde and 0.5% crystal for 30–60 min. Plates were scanned, and ImageJ software (version: 2.14.0/1.54f) was used to count colonies depending on mean area quantification.

### 4.9. Mice and Tumor Engraftment

C57BL/6J mice were purchased from Jackson Laboratory. Four- to six-week-old male mice were hosted at the MSU facility for two weeks. YUMM cells of 75% confluency were trypsinized and washed with sterile PBS. About 1 × 10^6^ cells suspended in 100 μL of PBS were subcutaneously injected into a shaved flank. Mice were monitored for developing palpable-sized tumors. Tumor size was measured with a digital caliper every two days to calculate tumor volume using the formula (length × width^2^ × 0.5).

### 4.10. Statistical Analysis

Comparative analysis was performed by unpaired two-tailed *t*-tests. Dose–response curves were fit using nonlinear regression [log(agonist) vs. response–variable slope (four parameters)]. Log-rank test was used for the analysis of mice survival. Data are presented as mean ± SEM, and a *p*-value < 0.05 was considered statistically significant. Statistical analyses were performed using the GraphPad Prism 10 software (La Jolla, CA, USA).

## Data Availability

Primary research data are available upon request to the authors. There are no large data sets appropriate for submission to repositories.

## Supplementary Materials

The supporting information can be downloaded at: https://www.mdpi.com/article/10.3390/ijms241813785/s1.

## Abbreviations

YUMM	Yale University Mouse Melanoma
YUMMER	YUMM exposed to radiation
Vem	Vemurafenib
MRTF	Myocardin related transcription factor
MAPK	Mitogen-activated protein kinase
ICIs	Immune checkpoint inhibitor proteins
ICT	Immune checkpoint therapy
IC_50_	half maximal inhibitory concentration
SRF	Serum response factor
