# Supplementary material for: Role of Rho/MRTF in Aggressive Vemurafenib-Resistant Murine Melanomas and Immune Checkpoint Upregulation
Source: Int J Mol Sci. 2023 Sep 7;24(18):13785. doi: 10.3390/ijms241813785 (PMC10531039; doi:10.3390/ijms241813785)
Supplement: Supplementary file 1 [file ijms-24-13785-s001.zip › ijms-2570153-supplementary.pdf]

Supplementary Table S1: Response of different YUMM cells to vemurafenib treatment.

		IC$_{50}$ of Vemurafenib (μM)		
**Cell line name**	**Genotype**	**P**	**R**	**Fold change***
**YUMM1.7**	$Braf^{V600E/wt}$ $Pten^{-/-}$ $Cdkn2^{-/-}$	0.4	10	33 ± 6.7
**YUMMER**	$Braf^{V600E/wt}$ $Pten^{-/-}$ $Cdkn2^{-/-}$	0.2	5.1	23 ± 5.7
**YUMM3.3**	$Braf^{V600E/wt}$ $Cdkn2^{-/-}$	0.7	5.7	8.0 ± 3.4
**YUMM5.2**	$Braf^{V600E/wt}$ $P53^{-/-}$	2.8	14.4	5.0 ± 1.0
**YUMM1G.1**	$Braf^{V600E/wt}$ $Pten^{-/-}$ $Cdkn2^{-/-}$ $Mc1r^{e/e}$	1.9	1.8	-
**YUMM4.1****	$Pten^{-/-}$ $Cdkn2^{-/-}$	34	34	-

P, Parental; R, Resistant
*, Fold change is the mean of change in IC$_{50}$ of the biological replicates
**, BRAF^{WT}

Supplementary Table S2 Fitted inhibition curve parameter values for Vemurafenib with varying concentrations of CCG-25708. Non-linear least squares fitted parameters for Vemurafenib concentration response curves in the presence of CCG-257081 are shown. The data were fitted in Graph Pad Prism 9.0 to a three-parameter model (i.e., Hill slope constrained to 1.0) with Bottom values constrained to be ≥ 0.

	**[CCG-257081] Parameter**	0 µM	0.45 µM	0.9 µM	1.8 µM	3.75 µM	7.5 µM	15 µM
**YUMM1.7_P**	Bottom	0.47	0.51	0.53	0.55	0.48	0.23	0.11
	Top	1.09	1.13	1.12	1.13	0.96	0.79	0.63
	LogIC50	-6.46	-6.74	-6.67	-6.88	-6.40	-5.81	-5.59
	IC50	3.47E-07	1.83E-07	2.15E-07	1.31E-07	4.02E-07	1.54E-06	2.57E-06
	Span	0.62	0.62	0.60	0.58	0.48	0.56	0.52
**YUMM1.7_R**	Bottom	0.00	0.00	0.00	0.00	0.00	0.29	0.47
	Top	1.06	1.02	1.05	1.01	0.91	0.72	0.62
	LogIC50	-4.61	-5.05	-4.81	-4.97	-4.97	-5.55	-7.04
	IC50	2.45E-05	9.03E-06	1.57E-05	1.08E-05	1.07E-05	2.84E-06	9.15E-08
	Span	1.06	1.02	1.05	1.01	0.91	0.43	0.15
**YUMMER_P**	Bottom	0.41	0.43	0.45	0.48	0.43	0.21	0.17
	Top	1.08	1.10	1.12	1.11	0.97	0.73	0.59
	LogIC50	-6.52	-6.86	-6.94	-7.09	-6.95	-6.12	-5.95
	IC50	3.05E-07	1.37E-07	1.14E-07	8.06E-08	1.11E-07	7.64E-07	1.12E-06
	Span	0.67	0.67	0.67	0.63	0.54	0.52	0.42
**YUMMER_R**	Bottom	0.01	0.16	0.21	0.25	0.22	0.15	0.25
	Top	1.07	1.06	1.09	1.07	0.99	0.72	0.50
	LogIC50	-5.20	-5.52	-5.57	-5.61	-5.59	-5.53	-5.32
	IC50	6.25E-06	3.01E-06	2.68E-06	2.49E-06	2.60E-06	2.94E-06	4.74E-06
	Span	1.07	0.90	0.88	0.82	0.78	0.56	0.25

Supplementary Figure Legends

Supplementary Figure S1: Resistant YUMM cells have increased surface area and Expression levels of Rho proteins. a: Vem-sensitivity of parental $BRAF^{V600E}$ cell lines (YUMM1.7 and YUMMER) and $BRAF^{Wildtype}$ (YUMM4.1). Cells were treated with increasing concentrations of Vem for 72 hours in DMEM with 10% FBS. An ATP-based assay (see Materials and Methods) was used to calculate cell viability relative to DMSO-treated cells. **b:** The surface area of YUMM **c**ells: Fixed cells were stained with vimentin, and the area was calculated using 40X objective microscopic images by ImageJ. *p < 0.05; ****p < 0.0001. **c-d:** Expression levels of Rho A (b) and Rho C proteins (c) were assessed by immunoblotting 30 µg total protein of whole cell lysates and normalized to β-tubulin. The results represent the mean ± SEM of four independent experiments. Graphs show normalized levels of Rho proteins of resistant cells calculated by ImageJ, and fold-change was calculated relative to YUMM1.7_P cells.

Supplementary Figure S2: Magnified Rhodamine phalloidin stained YUMM cells. Fixed cells were stained with Rhodamine Phalloidin. Images represent 1.5X magnification of different actin rearrangements: **a**: Enrichment of actin stress fibers, **b-c**: Cells displayed with cortical actin and depleted of actin stress fibers.

Supplementary Figure S3: Nuclear enrichment of MRTF-A. Western blot analysis of MRTF-A cytoplasmic fraction (C) and nuclear fraction (N) of indicated cell lines; P, parental; R, resistant. About 20 µg protein was loaded for each fraction. β-tubulin served as a cytoplasmic marker; histone H3 was used as a nuclear marker.

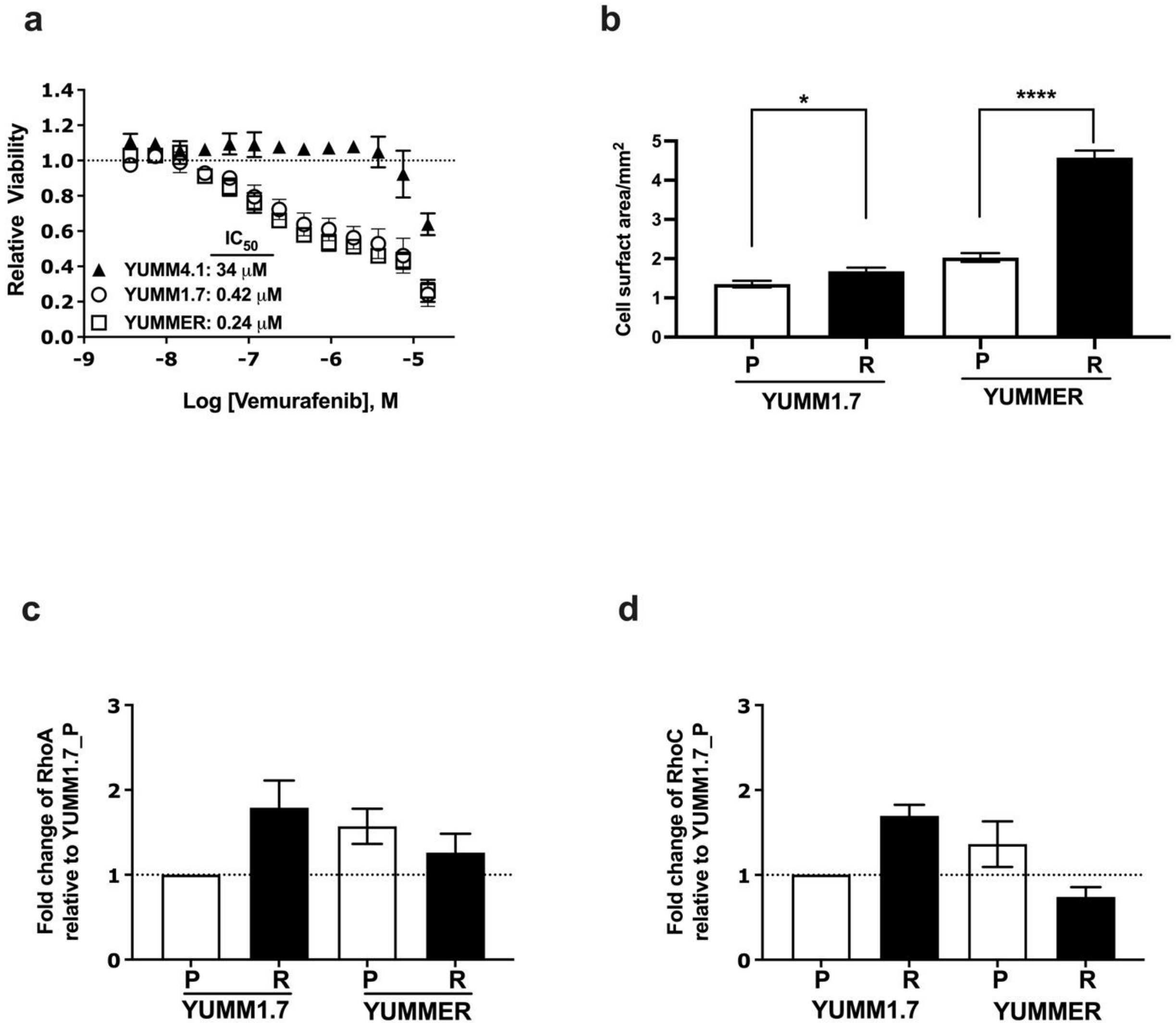

Supplementary Figure S1

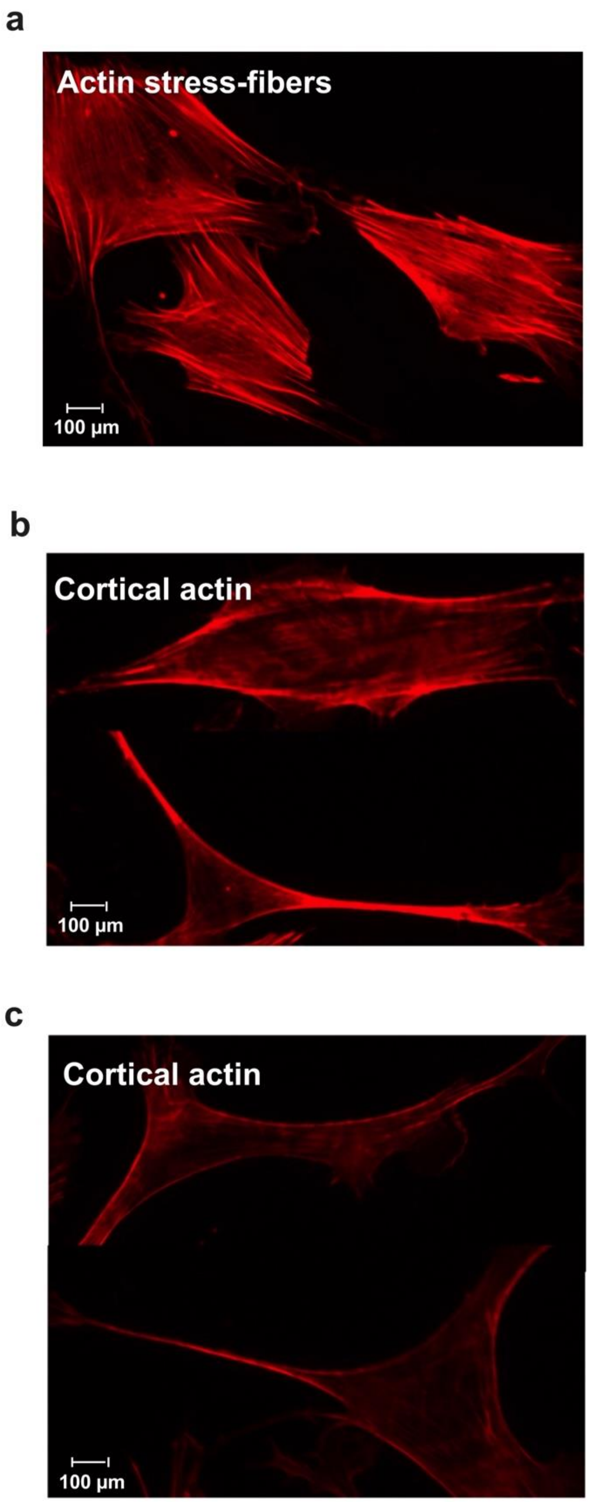

Supplementary Figure S2

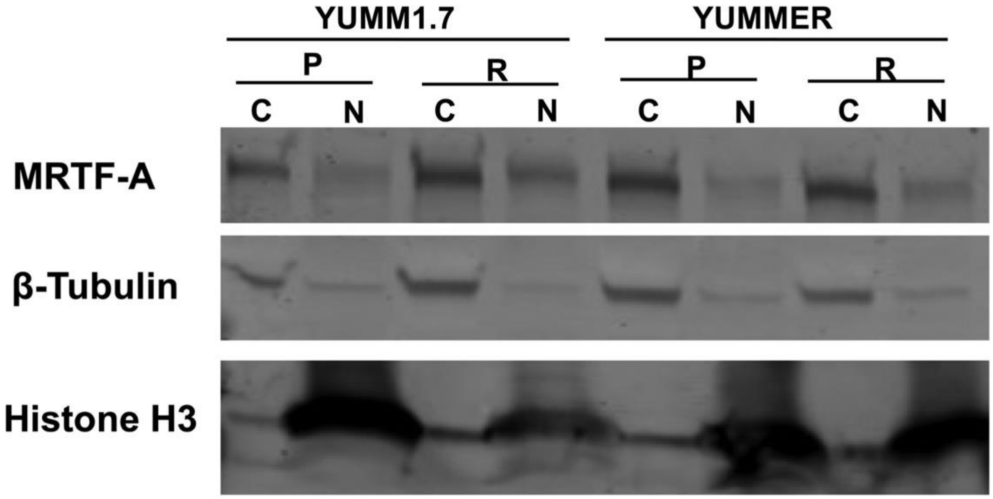

Supplementary Figure S3
